# Cross-Polarization Optical Coherence Tomography for Brain Tumor Imaging

**DOI:** 10.3389/fonc.2019.00201

**Published:** 2019-04-02

**Authors:** Konstantin S. Yashin, Elena B. Kiseleva, Ekaterina V. Gubarkova, Alexander A. Moiseev, Sergey S. Kuznetsov, Pavel A. Shilyagin, Grigory V. Gelikonov, Igor A. Medyanik, Leonid Ya. Kravets, Alexander A. Potapov, Elena V. Zagaynova, Natalia D. Gladkova

**Affiliations:** ^1^Microneurosurgery Group, University Clinic, Privolzhsky Research Medical University, Nizhny Novgorod, Russia; ^2^Laboratory of Optical Coherence Tomography, Research Institute of Experimental Oncology and Biomedical Technologies, Privolzhsky Research Medical University, Nizhny Novgorod, Russia; ^3^Laboratory of High-Sensitivity Optical Measurements, Institute of Applied Physics, Russian Academy of Sciences, Nizhny Novgorod, Russia; ^4^Department of Anatomical Pathology, Privolzhsky Research Medical University, Nizhny Novgorod, Russia; ^5^Federal State Autonomous Institution “N.N. Burdenko National Scientific and Practical Center for Neurosurgery” of the Ministry of Healthcare of the Russian Federation, Moscow, Russia; ^6^Research Institute of Experimental Oncology and Biomedical Technologies, Privolzhsky Research Medical University, Nizhny Novgorod, Russia

**Keywords:** cross-polarization optical coherence tomography (CP OCT), malignant brain tumors, glioblastoma, intraoperative imaging, imaging assessment

## Abstract

This paper considers valuable visual assessment criteria for distinguishing between tumorous and non-tumorous tissues, intraoperatively, using cross-polarization OCT (CP OCT)—OCT with a functional extension, that enables detection of the polarization properties of the tissues in addition to their conventional light scattering.

**Materials and Methods:** The study was performed on 176 *ex vivo* human specimens obtained from 30 glioma patients. To measure the degree to which the typical parameters of CP OCT images can be matched to the actual histology, 100 images of tumors and white matter were selected for visual analysis to be undertaken by three “single-blinded” investigators. An evaluation of the inter-rater reliability between the investigators was performed. Application of the identified visual CP OCT criteria for intraoperative use was performed during brain tumor resection in 17 patients.

**Results:** The CP OCT image parameters that can typically be used for visual assessment were separated: (1) signal intensity; (2) homogeneity of intensity; (3) attenuation rate; (4) uniformity of attenuation. The degree of match between the CP OCT images and the histology of the specimens was significant for the parameters “signal intensity” in both polarizations, and “homogeneity of intensity” as well as the “uniformity of attenuation” in co-polarization. A test based on the identified criteria showed a diagnostic accuracy of 87–88%. Intraoperative *in vivo* CP OCT images of white matter and tumors have similar signals to *ex vivo* ones, whereas the cortex *in vivo* is characterized by indicative vertical striations arising from the “shadows” of the blood vessels; these are not seen in *ex vivo* images or in the case of tumor invasion.

**Conclusion:** Visual assessment of CP OCT images enables tumorous and non-tumorous tissues to be distinguished. The most powerful aspect of CP OCT images that can be used as a criterion for differentiation between tumorous tissue and white matter is the signal intensity. In distinguishing white matter from tumors the diagnostic accuracy using the identified visual CP OCT criteria was 87–88%. As the CP OCT data is easily associated with intraoperative neurophysiological and neuronavigation findings this can provide valuable complementary information for the neurosurgeon tumor resection.

## Introduction

Optical coherence tomography (OCT) is a label-free, real-time imaging technique that allows three-dimensional images of biological tissues to be obtained at high resolution (around 2 μm). OCT is similar to ultrasonic imaging, in that both techniques detect reflected waves (light or acoustic). However, OCT uses a near-infrared light source (in the 700–1,300 nm wavelength range). OCT is a promising method for intraoperative guidance during the resection of glial tumors (astrocytomas) (1–3).

Recently, OCT has been proposed for intraoperative use in distinguishing tumorous and non-tumorous tissues using handled probes (4, 5) or microscope-integrated OCT systems (6, 7). OCT can provide differentiation between tumorous and non-tumorous tissues through both quantitative (4, 8) and qualitative (9, 10) assessment of the OCT signals. However, although visual assessment of the OCT data provided by clinically approved systems seems to be less sensitive when compared with the calculation of optical coefficients (which is not approved for clinical use), it is more user-friendly and intelligible. Meanwhile, for the intraoperative application of OCT it is necessary clearly to define the visual assessment criteria required for the OCT images in order to provide precise differentiation between glioma tissue and white matter.

Conventional, intensity-based OCT has demonstrated impressive results in detecting pathological changes in stratified tissues, such as those in the eye. However, the advanced visualization of structureless tissue types (brain, breast) needs novel contrast mechanisms such as can be achieved by using a so-called functional extension of OCT—polarization-sensitive (PS) OCT (11). PS OCT can detect the polarization state changes of the probing light in the tissue and, by means of this, generate tissue-specific contrast (11, 12). Based on the birefringence of the tissue structure, PS OCT provides better visualization of elongated structures and therefore provides advanced imaging of myelinated nerve fibers in nerves and the brain (13, 14), even showing the orientation of white matter tracts (15, 16). Cross-polarization OCT (CP OCT) is a variant of PS OCT that allows imaging of the initial polarization state changes both due to birefringence and cross-scattering in biological tissues (17, 18). In CP OCT two co-registered images are recorded: parallel (conventional OCT image or image in co-polarization) and orthogonal (image in cross-polarization) that detects tissue reflections with polarization state orthogonal to the incident one. Only orthogonally polarized backscattered light which is mutually coherent with the incident is contributing to the cross-polarized OCT image. The origin of such “coherent backscattering” includes random polarization during light propagation in the media, depolarization during the backscattering process, and “regular” polarization changes associated with propagation back and forth in birefringent media (19).

Some studies have demonstrated that tumorous tissue and white matter can be differentiated by visual assessment of such OCT images (8–10). This paper presents criteria based on the results of the CP OCT study of *ex vivo* specimens of human brain samples (20), compared with *in vivo* CP OCT images collected during brain tumor resections.

## Materials and Methods

This translational research was aimed to discover the CP OCT visual criteria for distinguishing timorous and non-tumorous tissue during surgical removal of a glioma. The study consists of two consecutive stages. During first part the *ex vivo* CP OCT analysis of brain human biopsy specimens matched to the actual histology was performed. Based on this data the visual criteria for distinguishing white matter and timorous tissue was discovered. The task of the second stage of the study was to develop the method of intraoperative using of the CP OCT and to confirm the discovered criteria during glioma removal.

### Patients

#### *Ex vivo* Study on Human Brain Specimens

**Figure 1:**
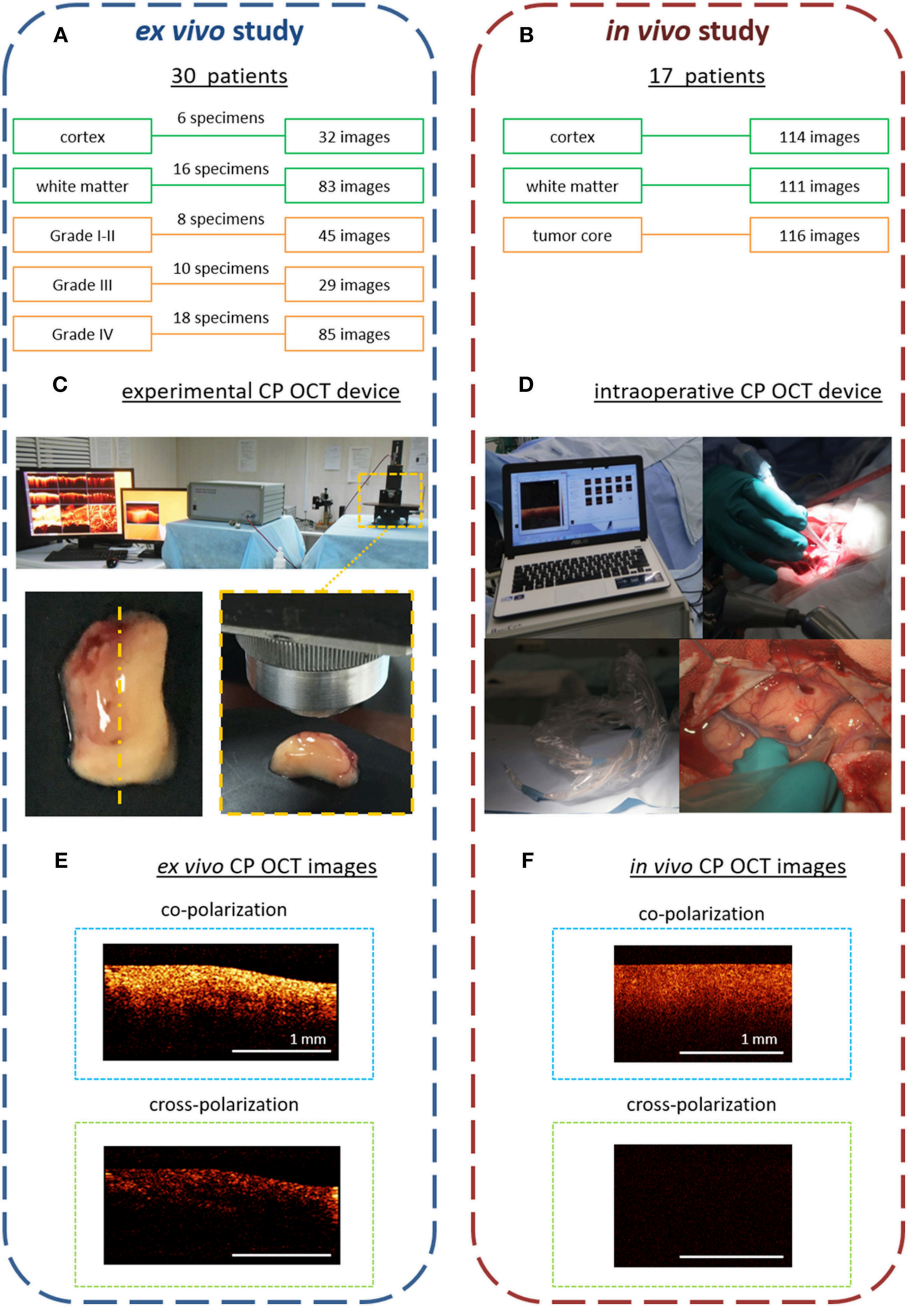
Design of the *ex vivo* and *in vivo* CP OCT study: **(A)** the *ex vivo* study was performed on material from operative biopsies: 30 patients with gliomas of different grades of malignancy; in total 274 *ex vivo* images were analyzed; **(B)** the *in vivo* study was performed on 17 patients with different grades of malignant brain tumors; in total 341 *in vivo* images were analyzed; **(C)** working area for CP OCT scanning with the experimental CP OCT device and on-mount optical probe; specimen with schematic marking of the scanning area along the central line (yellow dotted line); **(D)** CP OCT device approved for clinical use, with a handled OCT probe in a sterile cover; **(F)** *in vivo*, and **(E)** *ex vivo* CP OCT images in co- and cross-polarizations. The signal in cross-polarized image is orthogonally polarized backscattered light, which is mutually coherent with the incident one and can appear if the tissue has anisotropic structures such as myelinated fibers.

The *ex vivo* study was performed on material from human brain specimens that had been obtained during tumor resection from 30 patients with gliomas of differing degrees of malignancy: astrocytoma Grades I-II (*n* = 8), astrocytoma Grade III (*n* = 7), and glioblastoma Grade IV (*n* = 15) (Figure 1A). The tumor resections were performed taking into account eloquent brain areas and white matter tracts using a frameless navigation system with uploaded functional MRI data and intraoperative neurophysiological monitoring (also “awake” surgery). The surrounding tumor white matter in the peritumoral area that was routinely subjected to coagulation was accurately marked and removed. Samples were taken from different parts of each tumor.

The removed specimens were immediately placed in Petri dishes and closed to prevent dehydration. Tissues also were kept on ice until transfer to the imaging stage. Before OCT imaging, the tissue surface was cut to create a flat fresh surface of the sample. The CP OCT study of each sample was no longer than 30 min (including tissue preparation). In total, 176 samples of different tissue types were studied and 274 *ex vivo* images were obtained. After surgery, any worsening in neurologic state of patients was not recorded.

#### Intraoperative Study

Here, *in vivo* CP OCT images were collected during tumor resections in 17 patients with different malignant brain tumors: astrocytoma Grades I-II (*n* = 5), astrocytoma Grade III (*n* = 9), glioblastoma Grade IV (*n* = 2), breast cancer metastasis (*n* = 1). During the tumor resections the *in vivo* OCT images were collected using an approved CP OCT device with a handled probe enclosed in a sterile cover. In total, 341 images of three areas of interest were analyzed: cortex−114, white matter−111, tumor core−116 (Figure 1B).

This study was carried out in accordance with the recommendations of the World Medical Association's Declaration of Helsinki. The protocol was approved by the Ethical Committee of the Privolzhskiy Federal Research Medical Center of the Ministry of Health of the Russian Federation. All subjects gave written informed consent in accordance with the Declaration of Helsinki. All studies were performed in accordance with the relevant guidelines and regulations.

### Cross-Polarization OCT Devices

The *ex vivo* studies were performed with an multimodal OCT device with cross-polarization detection developed by the Institute of Applied Physics of the Russian Academy of Sciences (Nizhny Novgorod, Russia) (21, 22). The device operates at a central wavelength of 1.3 μm providing axial and lateral resolutions, in air, of 10 and 15 μm, respectively. The probing beam uses circular polarization. The device has a scanning rate of 20,000 A-scans/s and performs 2D lateral scanning within a range of 2.4 × 2.4 mm^2^ to obtain the 3D distribution of backscattered light in polarizations parallel and orthogonal to the polarization of the probing beam. Thus, the resulting CP OCT image includes an upper part—co-polarization image and a lower part—cross-polarization image. Scanning was performed in contactless mode (Figure 1C).

For *in vivo* study, time-domain “Polarization-sensitive optical coherence tomograph OCT-1300U” (BioMedTech LLC, Nizhny Novgorod, Russia) was used (Figure 1D). It is approved for clinical use (product license FCP 2012/13479 from 30 May 2012) has the same characteristics of laser radiation as the experimental CP OCT device. However, its image data-processing system is not as effective, so the intensity of the intraoperative images in co- and cross-polarization is lower (~4 times) (Figure 1F) compared to those obtained using the experimental CP OCT device (Figure 1E).

### Histological Study

After imaging the scanning area on the specimen was marked with histological ink, then the specimen was fixed in 10% formalin for 48 h and re-sectioned through the marked area, so that the plane of the histological sections coincided to en-face the CP OCT images. For the histological evaluation, hematoxylin and eosin staining was used. Two histopathologists independently evaluated the histological slides, with their diagnoses coinciding in 98% of cases.

### Visual Assessment of CP OCT Images

Based on an initial analysis of the *ex vivo* CP OCT images of the white matter and tumors the parameters of the CP OCT signal for further analysis were selected. The differential criteria for CP OCT images must satisfy the following conditions:

- Simplicity and high speed of evaluation (the signal indicator(s) should be as simple as possible in use, and easy for the operating surgeon to remember);
- Be informative (reflect the histological morphology of the tissue);
- Have a high degree of inter-expert reliability (i.e., not resulting in significant disagreements in interpretation).

Visual assessment of the CP OCT images was performed using two special tests containing a training set and a proper test of 100 images (26 images of white matter and 74 images of tumorous tissue: Grade II−12, Grade III−22, Grade IV−40). The CP OCT images selected for these tests corresponded to specimens with typical histological structures of the tumor and white matter (cropped and damaged images, and images that, according to the histological samples contained large necrotic or hematoma areas were excluded). The first test, aimed at identifying the most useful CP OCT criteria, contained OCT images separately in co- and cross-polarization; the second test, aimed at determining the diagnostic accuracy of distinguishing tumor and white matter, contained OCT images separately in co- and cross-polarization, and simultaneously in both polarizations. Each test was performed by three “blinded” investigators.

**Figure 2:**
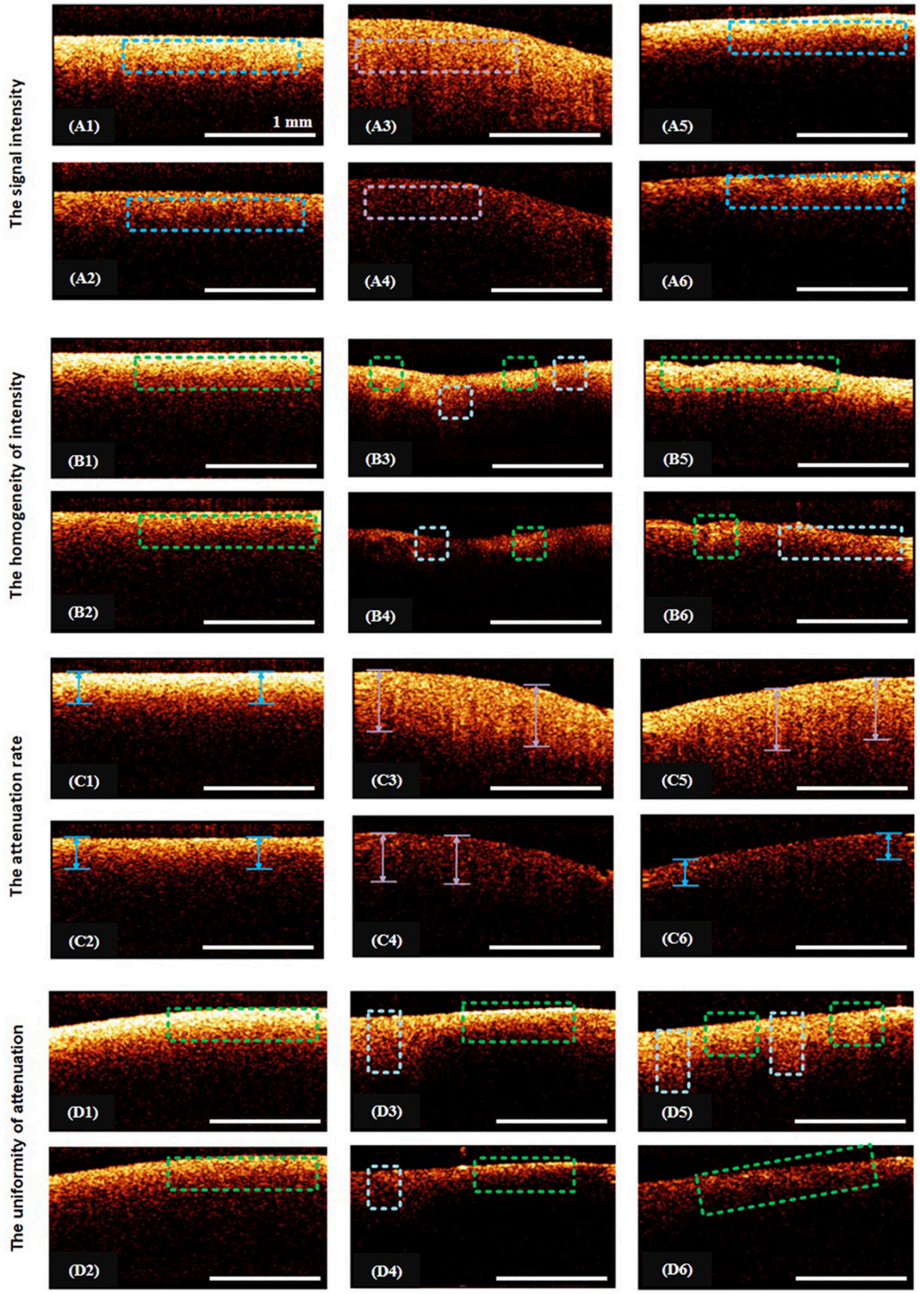
Illustration from the training set of CP OCT images: **(A1–A6)**, **(B1–B6)**, **(C1–C6)**, **(D1–D6)** show examples of the assignment of certain characteristic to the CP OCT signal; **(A1–A6)**—intense signals are marked with blue rectangles, violet—a low-intensity signal; **(B1,B2)**—the regions of homogeneous signals are indicated by green rectangles; **(B3–B6)**—green areas and pale blue squares indicate areas of different intensities; **(C1–C6)**—blue arrows denote regions with high rates of signal attenuation, violet—with low signal attenuation rates; **(D1,D2,D6)**—the green rectangles indicate areas with uniform attenuation of the signal; **(D3–D5)**—rectangles of pale blue and green color indicate regions with different signal attenuation rates.

In the first test, the visual assessment of the CP OCT image was performed on the basis of the following parameters (Figure 2) each with only two possible alternatives, which were selected to satisfy the condition of simplicity and high speed of evaluation (the indicator should be as simple as possible in use, and easy to remember by the operating surgeon):

1. The signal intensity (“intense”/“non-intense”)—the average level of the OCT signal throughout the image.
2. The homogeneity of intensity (“homogeneous”/“heterogeneous”)—the lack of variability of the brightness of the OCT signal.
3. The attenuation rate (“high”/“low”) as estimated by the penetration depth of the probing radiation;
4. The uniformity of attenuation (“uniform”/“non-uniform”)—the uniformity of the OCT signal attenuation along the inferior border of the structural OCT image.

The extent to which the parameters were “informative” was identified based on measuring the degree of association of each visual parameter with the results of the histology during visual assessment by the three “blinded” investigators, between whom the degree of inter-expert reliability was also identified.

**Figure 3:**
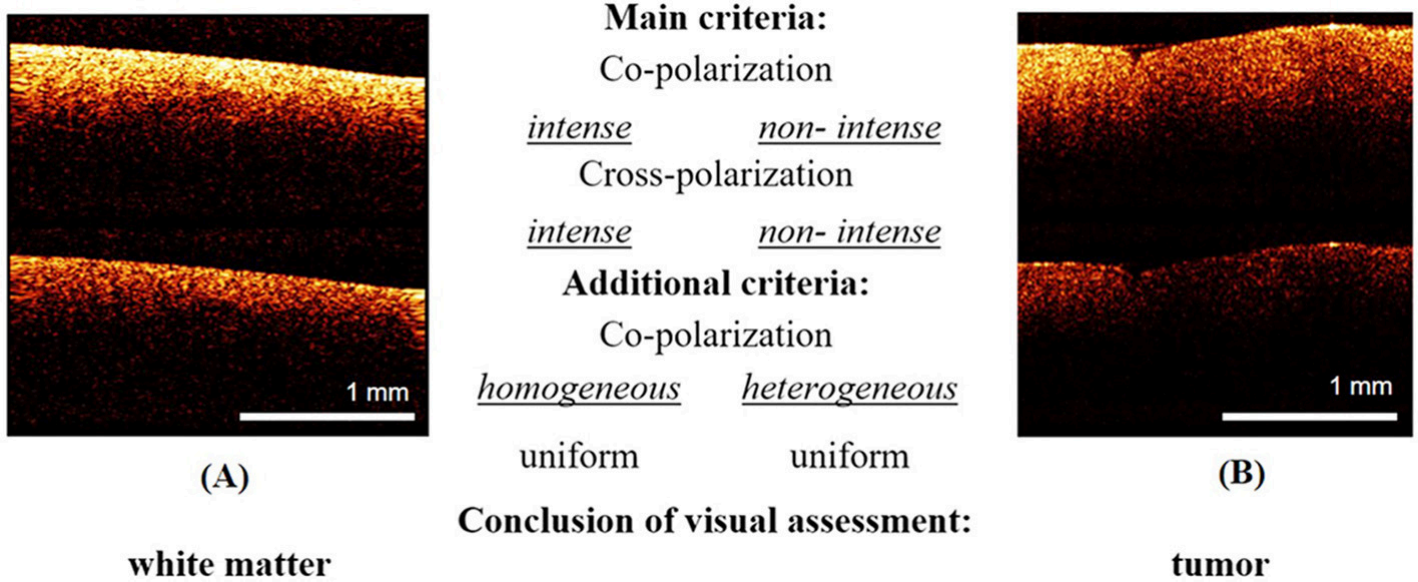
Examples of images from the training set for the second test: **(A)**—white matter, **(B)**—tumor. The responder identifies tissue type using main criteria: white matter is characterized by high intense signal in co- and cross-polarization unlike low intensity signal of tumorous tissue. In the doubtful case, the additional criteria can be used.

The second test was also performed by three “blinded” investigators (neurosurgeons) and was based on the main and additional visual criteria identified during the first test. There were two possible answers: “tumor”/“white matter” (Figure 3). The inter-rater reliability between the investigators was also recorded.

### Statistical Analysis

The statistical analysis was performed using Statistica 10.0, IBM SPSS Statistics 20. For evaluation of the association ratio of the coefficients Q (Yule) and φ (phi) were used, with a statistically significant value for *Q* ≥ 0.5 and for φ > 0.3. The inter-rater reliability between investigators was registered using the Fleiss' kappa (κ) and Krippendorff's alpha (α) coefficients: κ (α) ≥ 0.8—perfect agreement; 0.7 ≤ κ (α) < 0.8—substantial agreement; κ (α) < 0.7—poor agreement. For the second test the diagnostic test parameters (sensitivity, specificity and accuracy) were calculated.

### Intraoperative Application of the CP OCT

There was no histopathological evaluation of the *in vivo* CP OCT images, for error prevention and checking of complementarity between the OCT image and tissue type, the scanning being performed in areas with no doubt about their histology (white matter and cortex far from the tumor mass, and of regions within the tumor core) using image guiding under a neuronavigation system and high magnification of surgical microscope. The *in vivo* CP OCT data were compared with the *ex vivo* set and also combined with surgical microscope view, the preoperative MRI and neuronavigation data, intraoperative neurophysiological and neuronavigation findings.

## Results

### *Ex vivo* CP OCT Images of White Matter and Tumorous Tissue

The CP OCT signal of the *ex vivo* specimens is more intense and has a higher attenuation rate (in both the initial and orthogonal polarizations) than those obtained *in vivo*. However, previous comparative analysis of the optical properties of white matter and tumorous tissues has demonstrated that the CP OCT images obtained *ex vivo* show full qualitative similarity to the *in vivo* CP OCT images (23). For the cortex there are also structural differences between the *ex vivo* and *in vivo* images (23). In the *in vivo* studies, the CP OCT images show a specific vertical striation arising from “shadows” of the blood vessels located just under the tissue surface (**Figure 5A1**); this striation is practically invisible on the *ex vivo* images due to vasoconstriction.

#### White Matter

**Figure 4:**
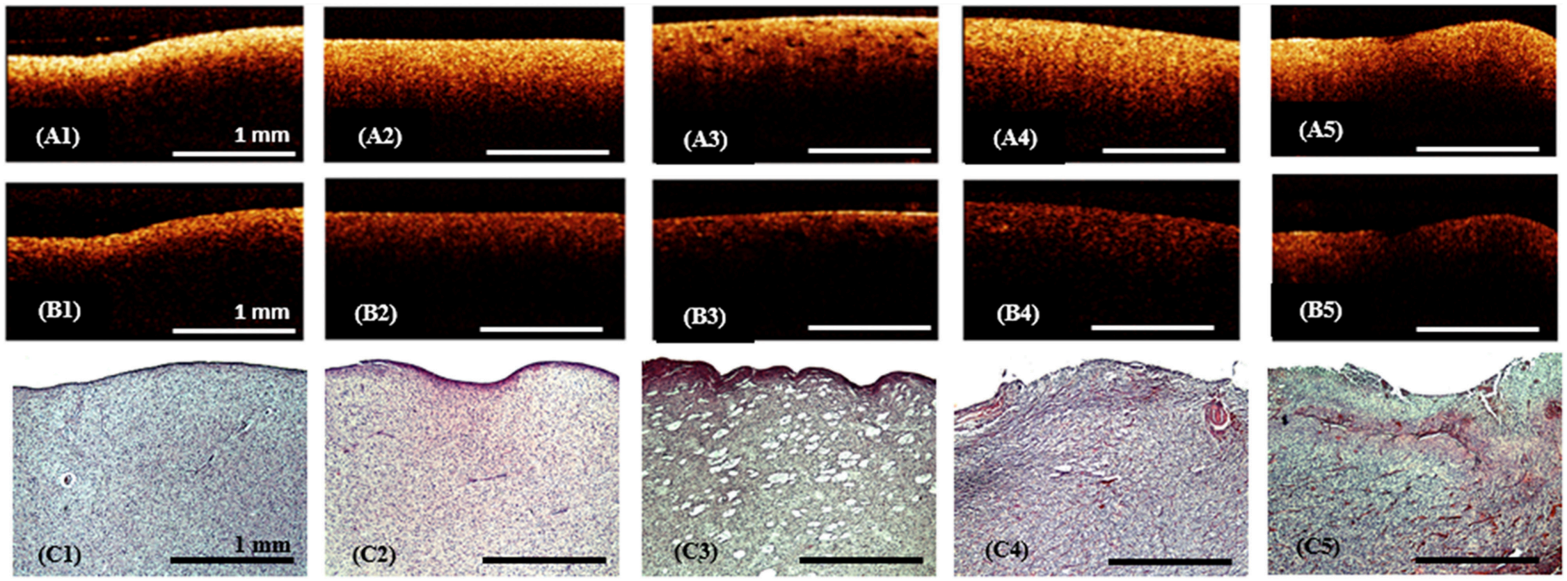
*Ex vivo* CP OCT images **(A1–A5)**, **(B1–B5)** and corresponding histology **(C1–C5)** of white matter **(A1–C1)**, diffuse astrocytoma Grade II **(A2–C2)**, **(A3–C3)**, diffuse astrocytoma Grade III **(A4–C4)** and glioblastoma **(A5–C5)**; on the CP OCT image of diffuse astrocytoma Grade II can be seen microcysts typical for this type of tumor **(A3,C3)**; **(A1–A5)**—CP OCT images in co-polarization and **(B1–B5)**—in cross-polarization. Histological images **(C1–C5)**-hematoxylin and eosin staining.

CP OCY analysis of *ex vivo* specimens showed that the white matter on the CP OCT images in co- and cross-polarizations (Figures 4A2,B2) presented by a narrow stripe of high intensity OCT signal. Histologically, the white matter is represented by a regular and dense arrangement of myelinated fibers (Figure 4C1) and therefore is characterized by high scattering properties. This explains the presence of a high-intensity homogeneous but rapidly decaying OCT signal in both co- and cross-polarization.

#### Gliomas

In gliomas the tissue elements are discohesive and disordered (Figures 4C2–C4); although there are a few elements with high-scattering properties (Figure 4C5). Therefore, the CP OCT images of tumorous tissue are characterized by low signal intensities in co- (Figures 4A2–A5) and cross-polarizations (Figures 4B2–B5). Grades I-III astrocytomas on the CP OCT images are represented by low intensity and slowly attenuating signals (Figures 4A2–A4,B2–B4), however the signal may be homogeneous (Figures 4A2,A4,B2,B4) or heterogeneous in the presence of cysts, calcification, and hemorrhaging (Figures 4A3,B3). The CP OCT signal of a glioblastoma is of low intensity, and heterogeneous with tessellated areas of high intensity (Figures 4A5,B5) corresponding to areas of high cell density, necrosis or hemorrhaging (Figure 4C5). The attenuation rate of the CP OCT signal may be high or low with the attenuation along the inferior border of the image being uniform or non-uniform.

The initial comparative analysis of CP OCT images of brain specimens with typical histological structures has demonstrated the capability of OCT to differentiate between white matter and tumorous tissue. However, there are no clear criteria for distinguishing between white matter and tumorous tissue due to the variability of the signal characteristics of the gliomas. Therefore, it is also evident that OCT cannot be used for the intraoperative grading of gliomas.

### Visual Assessment Criteria for CP OCT Images for Distinguishing Between White Matter and Gliomal Tissue

Between all investigators the values of the coefficients Q and φ were high for the parameter “signal intensity” in co- and cross-polarization (Qco = 0.91–0.92; φco = 0.64–0.65; Qcross = 0.92–0.94; φcross = 0.64–0.70) and also statistically significant for the parameters “the homogeneity of intensity” (Qco = 0.86–0.94; φco = 0.47–0.50) and “the uniformity of attenuation” (Qco = 0.58–0.82; φco = 0.30–0.44) in co-polarization. The inter-rater reliability between investigators was perfect for the parameter “signal intensity” in both polarizations (κ = 0.90; α = 0.90) and substantial for “the homogeneity of intensity” (κ = 0.79; α = 0.79) and “the uniformity of attenuation” (κ = 0.75; α = 0.75) in co-polarization (Table 1).

**Table 1:** Visual assessment criteria of CP OCT images for distinguishing between white matter and glioma tissue.

**CP OCT image parameter**	**White matter**	**Tumor**
**MAIN CRITERIA**		
The signal intensity in co-polarization	*Intense*	*Non-intense*
The signal intensity in cross-polarization	*Intense*	*Non-intense*
**ADDITIONAL CRITERIA**		
The homogeneity of intensity in co-polarization	Homogeneous	Heterogeneous
The uniformity of attenuation in co-polarization	Uniform	Un-uniform

Based on the results of the first test the most powerful criteria for the visual assessment of microstructural CP OCT images to enable differentiation between glial tumor tissue and white matter are the CP OCT signal intensities in co- and cross-polarization. Also for this aim, the homogeneity of intensity and the uniformity of attenuation can be used as additional criteria.

### Diagnostic Accuracy of CP OCT Based on Visual Assessment of Images

The results of the second set of tests, using the identified main and additional criteria, separately in co- and cross-polarization and simultaneously in co- and cross-polarization (Table 2) demonstrate their great inter-rater reliability. The test based on simultaneously assessing CP OCT images in co- and cross-polarization showed a higher diagnostic accuracy (87–88%).

**Table 2:** The results of diagnostic test by visual assessment of the CP OCT images.

**Polarization**	**Inter-rater reliability rate**	**Sensitivity, %**	**Specificity, %**	**Diagnostic accuracy, %**
Co-	0.83/0.83	89–93	67–73	83–84
Cross-	0.86/0.86	80–87	75–89	82–83
Co- and cross- simultaneously	0.92/0.91	82–85	92–94	87–88

Using co-polarization showed higher sensitivity (89–93%); therefore using this regimen allows minimization of the risk of failure of tumor detection. The high specificity of 92–94% that can be achieved by using simultaneous visual assessment of the images in co- and cross-polarization is associated with the low risk of misguided white matter resection.

### Intraoperative Visual Assessment of the CP OCT Images

The intraoperative *in vivo* CP OCT images of white matter and tumors obtained by the certified OCT system with the handled probe show full structural similarity to the *ex vivo* CP OCT images of brain specimens obtained using the laboratory OCT setup, however, owing to differences in image processing between the OCT systems the intensity of the intraoperative images in co- and cross-polarization are lower (~4 times).

**Figure 5:**
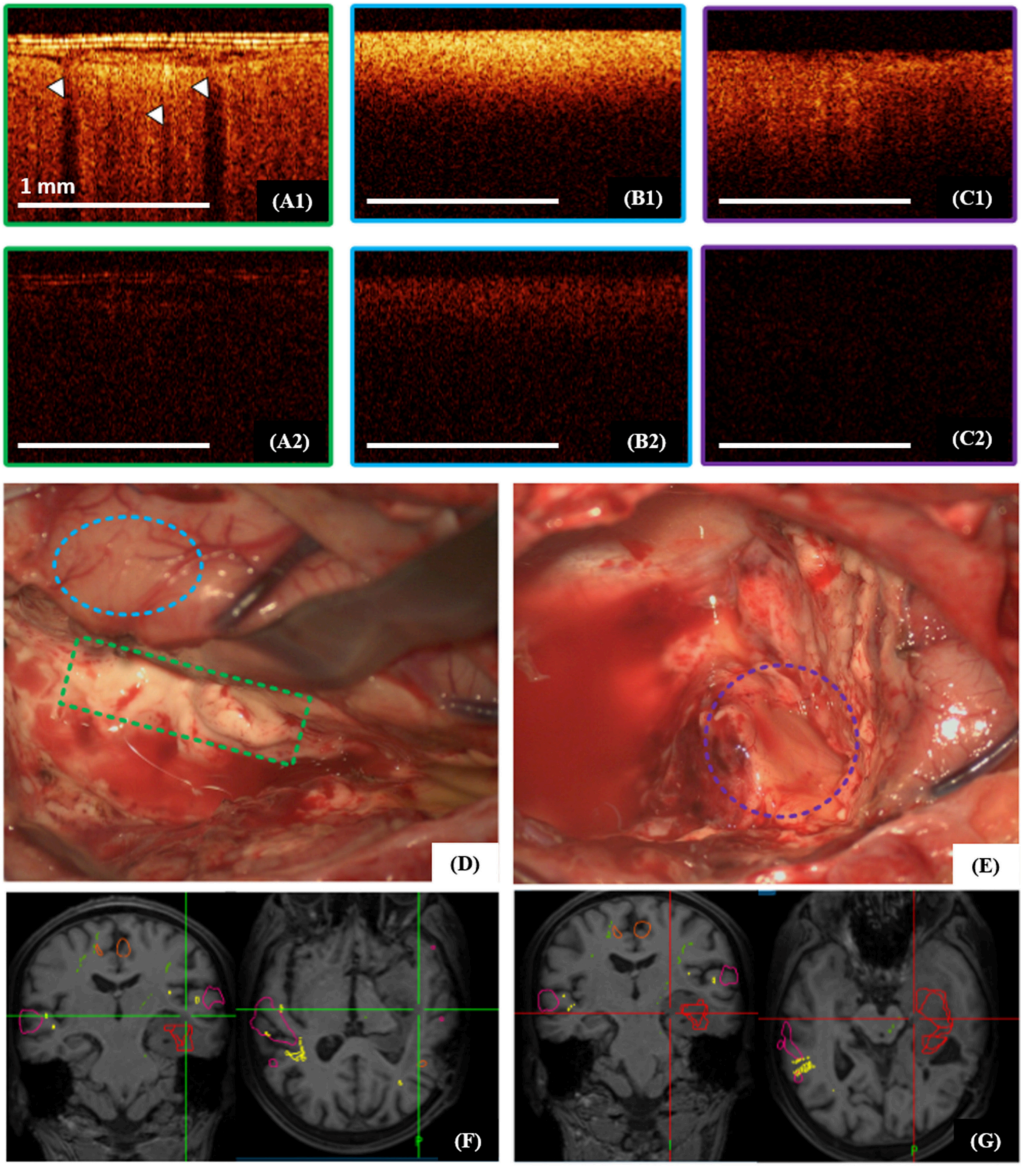
Intraoperative CP OCT images of cortex **(A1,A2)**, white matter **(B1,B2)** and diffuse astrocytoma Grade II **(C1,C2)**; the scanning areas of the corresponding tissue types in the surgical field are marked with green **(A1,A2)**, blue **(B1,B2)** and violet **(C1,C2)** dotted lines **(D,E)**, and interrelated with the neuronavigation data **(F,G)**; **(A1–C1)**—CP OCT images in co-polarization and **(A2–C2)**—in cross-polarization. The white arrows show characteristic vertical striations arising from “shadows” of the blood vessels located just under the tissue surface.

The cortex is characterized by specific vertical striations arising as the “shadows” of the blood vessels located just under the tissue surface (Figures 5A1, 6A1), and these striations can be considered as a specific cortex-indicator. The white matter tissue is characterized by its high attenuation, although the intensity is lower in cross-polarization than for the signal in co-polarization (Figures 5B1,B2, 6B1). Regardless of the glioma grade, the tumorous tissue is characterized by low-intensity, homogeneous or heterogeneous signals with low attenuation rate in co-polarization (Figures 5C1, 6A2,A3, images in co-polarization). Since the OCT device approved for clinical use only provides imaging at low intensity the signal in cross-polarization is therefore almost absent from the tumor (Figures 5C2, 6A2,A3, images in cross-polarization) and very low from the cortex (Figures 5A2, 6A1, images in cross-polarization). The scanning areas of the corresponding tissue types in the surgical field are marked with green (cortex, Figures 5A1,A2), blue (white matter, Figures 5B1,B2) and violet (diffuse astrocytoma Grade II, Figures 5C1,C2) dotted lines (Figures 5D,E) and interrelated with the neuronavigation data (Figures 5F,G).

**Figure 6:**
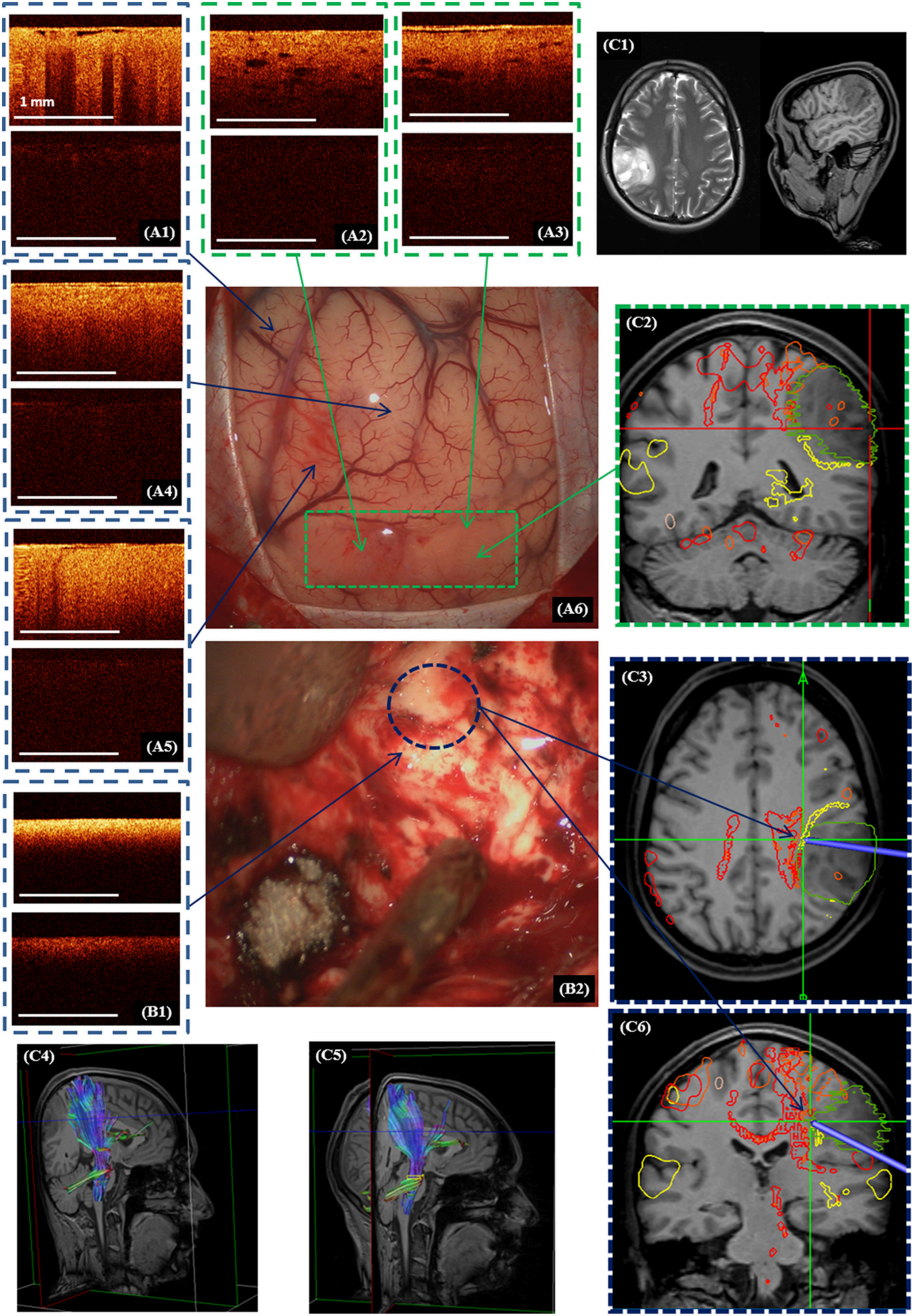
Intraoperative CP OCT images in a patient with diffuse astrocytoma (Grade II): of the cortex **(A1)** with well-defined (**A2,A3**; **A6**—green dotted line) and invisible **(A4,A5)** tumor invasion, white matter **(B1)** close to the right corticospinal tract (**B2**—blue color dotted line; **C3,C4**—marked by red color); the scanning areas of corresponding tissue types in the surgical field **(A6,B2)** are marked with green **(A6)** and, blue **(B2)** dotted lines and interrelated with the neuronavigation data, where the tumor is marked with a green line and the corticospinal tract with a red line **(C2,C3,C6)**. Preoperative MRI in T1 and T2 **(C1)** and the corticospinal tract reconstruction based on the DTI before **(C4)** and after **(C5)** operation. **(A1–A5,B1)**—CP OCT images in co-polarization (upper image) and cross-polarization (lower image).

OCT scanning of the cortex was performed in 9 cases (53%). In all the comparisons, the OCT and neuronavigation data showed that the tumor had widened through the tumor margin that can be detected under the white light of the operating microscope. In the case of visible cortical invasion by the tumor the OCT scanning was performed from this area to visually healthy brain tissue. In the case of visually intact cortex, the scanning was started from the central part of the tumor as detected by the neuronavigation system.

Taking into account the variability of the CP OCT images for a variety of different reasons (e.g., brain swelling), when distinguishing white matter and tumor it is better to commence the OCT scanning of white matter from a control area (where there is no doubt about the histology) to enable comparison of this scan with following images. In our study scanning of control area before tumor resection was performed only in 9 patients (53%), while in the other cases the identified criteria (Table 1) and standard CP OCT images of white matter (Figures 4A1,B1, 5B1,B2) were used.

Perfect detection of white matter by OCT can be useful when tumor resection is performed close to white matter tracts. Figure 6 shows the process of removal of a diffuse astrocytoma (Grade II) in the right parietal lobe. The preoperative diffusion tensor imaging (DTI) data uploaded in the neuronavigation system demonstrated that the tumor was closely adjacent to right cortico-spinal tract (Figures 6C2–C4). Electrical stimulation in this region had shown a response at a current of 5 mA (pulse frequency of 60 Hz, with a single phase duration of 1 ms in trains lasting 4 s), that meant the approximate distance to the tract was about 5 mm. Based on the identified criteria, OCT scanning demonstrated the presence of white matter in this region. It was taken decision to stop further resection. On post-operative DTI, no change in the corticospinal tract was detected. Therefore, the simultaneous use of subcortical electrostimulation and OCT was able to define more exactly the required extent of resection close to eloquent white matter tracts.

## Discussion

During surgery of infiltrative brain tumors the benefits of novel intraoperative imaging technologies are self-evident, due to the lack of resolving capacity of the operating microscope for distinguishing tumorous and non-tumorous tissues and the detection of any residual tumor (24–27). In order to overcome this problem, at present, the most commonly used technologies are fluorescent visualization with 5-ALA (28–30) and intraoperative MRI (31, 32). However, these methods have some limitations; therefore, the development of novel intraoperative technologies has become particularly relevant. OCT appears to provide a very promising method for routine neurosurgical practice due to the clear benefits of this method such as: safety (using a near infrared light source does not risk tissue damage); accuracy (high resolution ~10 micron) the absence of a requirement for contrast agents; imaging depths of more than 1 mm; the ability to obtain images at a distance and therefore the opportunity for integration into a surgical microscope or endoscope (2, 3).

Obviously, the increased interest in OCT has been the result of the continuing improvements in several aspects of OCT, such as imaging speed, sensitivity, the development of functional OCT extensions and signal data processing. The quantitative assessment of OCT with the determination of various optical coefficients such as backscattering (33) and attenuation (4, 10, 34) coefficients provides higher diagnostic accuracy compared with the qualitative assessment of OCT images. Kut et al. have demonstrated the high diagnostic accuracy of OCT for distinguishing tumors from white matter on the basis of the optical attenuation coefficient: for patients with higher-grade tumors the sensitivity/specificity reached 92%/100%; for low-grade tumors sensitivity/specificity values were 100%/80% (4). Unfortunately, at present, clinically approved OCT systems can provide only visual assessment, although that has been sufficient to improve readability for neurosurgeons. Additionally, such visual assessment for distinguishing white matter from tumor tissue also provides high sensitivity of (82–85%), specificity (92–94%), and diagnostic accuracy (87–88%). Therefore, it can be proposed for routine clinical use, although with some restrictions.

This paper deliberately references previously published data (20) with regard to intraoperative approval of the visual CP OCT criteria identified for distinguishing white matter from tumor tissue. These criteria have been specified in place of the previously suggested “loss of normal attenuation” from the tumorous tissue (9, 10). However, this criterion remains relevant for the detection of tumor invasion into the cortex, which is characterized by a “typical view” of vertical striations, disappearing in cases where there are any changes of cortical microarchitecture. The result of this study questions the significance of the “homogeneous” criterion for distinguishing tumor and white matter because of the variability of the characteristics of the OCT signal from gliomas. For example, tumorous tissue may have a quite homogeneous OCT signal, while, on the other hand, white matter in the vicinity of necrotic areas or hematomas can lose its homogeneity.

The study has shown that CP OCT, as an extension of OCT, broadens the functionality of the method. The assessment of CP OCT images simultaneously in co- and cross-polarization has shown a higher specificity (92–94% against 67–73%) and diagnostic accuracy (87–88% against 83–84%) compared with conventional OCT. Therefore, this mode is effective to use during tumor resection closely adjacent to eloquent white matter tracts. Using CP OCT, based on visual assessment along with co-polarization, shows high sensitivity (89–93% against 82–85% when using co- and cross-polarization simultaneously) and therefore it is effective in improving identification of the necessary extent of resection in non-eloquent brain areas.

In this study, discrete histological tissue types such as cortex, white matter and tumor have been described. The formation of the OCT signal and its differences between tumor and white matter can be explained using a simple model of the structural organization of the tissue: tumorous tissue can be represented as an assembly of tumor cells that are characterized by low attenuation, compared to the high attenuation of myelin. However, differentiation using visual assessment can be difficult when comparing white matter with structureless (or intact) myelin and tumorous tissue with necrosis (or necrosis alone), due to their similarity in optical properties. Tumorous tissue without necrosis forms the typical structure of all Grade I-III astrocytomas and the peripherial area of glioblastomas (Grade IV). Necrotic areas characterized by high attenuation (forward cross-scattering) always present in (1) the glioblastoma core, (2) tissues after radiotherapy (e.g., in the case of recurrent astrocytoma after combination treatment) and (3) tissues following bipolar coagulation (total necrosis). The high attenuation (forward cross-scattering) of white matter is the result of the high-density packaging of its myelin fibers. Some events such as structural damage of the myelin, or cerebral edema related to tumor growth, can lead to a significant decrease of attenuation and of forward cross-scattering (35). These myelin changes are typical of the peritumoral area of high-grade astrocytomas. Probably, improvements in the quantitative assessment of OCT data will allow these tissue types to be clearly distinguished.

At the moment, in all doubtful cases, consideration must be given to the patient‘s preoperative data, localization of the OCT scanning area, and the distance from eloquent brain areas. For example, if the preoperative clinical data is indicative of a low-grade glioma, during tumor resection no necrotic areas or myelin damage would be expected, so the surgeon would not expect to find necrotic areas or dramatic changes in the myelin structure, meaning that distinguishing between white matter and tumor is not an issue In the case of a glioblastoma, the white matter in the peritumoral area might be characterized by low attenuation and forward cross-scattering, so the intensity of the CP OCT signal could decrease in co- and cross-polarization. On the other hand in the case where there has been previous radiation therapy (e.g., recurrent glioblastoma) the tumor mass could include a vast amount of necrotic tissue and be characterized by high intensity in the CP OCT images. Therefore, in both cases the differentiation between white matter and tumorous tissue can be difficult. Consequently, in these doubtful cases, decisions about the extent of resection should primarily be based on the distance from eloquent brain areas. Hemorrhages can also compromise the interpretability of the OCT signals (10), therefore, before scanning, they should be removed together with any necrotic areas associated with bipolar coagulation.

In contrast to white matter, the cortex does not contain densely packed myelin fibers that might lead to difficulties in distinguishing between cortex and tumor, although tumor is characterized by higher cellularity and nuclear density comparing to cortex. Visual assessment of OCT signals based on the criterion “loss of normal attenuation” allows tumors to be distinguished from the cortex during *in vivo* scanning (10, 23). Probably, additional *in vivo* studies would enable clearer determination of the optical differences between cortex and tumor and offer better definitions for the quantitative OCT criteria (23).

This study was based on *ex vivo* brain specimens for a number of reasons: (1) the possibility of carrying out maximally targeted histological examinations to appropriately validate the OCT; (2) the absence of strict time limitations, which made it possible to perform multiple scans to monitor the image quality. The previously performed comparative analysis of the optical properties of white matter and tumorous tissues of the brain allow us to conclude that the CP OCT images obtained *ex vivo* show a full qualitative similarity to the *in vivo* CP OCT images (23). However, *ex vivo* study is not representative for the cortex by reason of the disappearance of the unique characteristics of the CP OCT signal in *ex vivo* CP OCT images.

The study has several limitations. One of them is the use of different CP OCT devices in the preclinical part and during the intraoperative scanning. Although the experimental device has some advantages due to a better signal characteristics and a more detailed computer processing of the OCT data, using it during surgical procedures was not possible due to its novel character and the absence of permission for its clinical use. Therefore, the study demonstrates the advantages of CP OCT on fresh pathological specimens, but not for intraoperative CP OCT data. In the *ex vivo* CP OCT study based on fresh specimens it is easier to “target” the region of interest and therefore provide advanced histological validation of CP OCT image in comparison with *in vivo* CP OCT study, where image interpretation can be complicated by the impact of several factors such as tissue deformation during its removal, difficulties in access to the region of interest where the white matter is infiltrated by tumor, active bleeding and use of hemostatic materials affecting the quality of the CP OCT images. However, the promising results of this study allow us to recommend implementing a clinical CP OCT prototype based on the experimental one used in this study.

Further, only three raters were tested, therefore the results may be considered as preliminary. Increasing the number of raters may improve the results in terms of visual assessment, diagnostic accuracy, sensitivity and specificity for CP OCT for distinguishing white matter from tumor tissue.

The limitation of the intraoperative phase of the study was lack of morphological verification of scanning areas. Using image guiding under neuronavigation system and high magnification of surgical microscope doesn‘t provide exact information about tissue morphology.

Nevertheless, while OCT does not provide molecular information and cannot detect individual tumor cells, it looks promising in assisting navigation during neurosurgical procedures such as glioma resection, stereotactic biopsy and the placement of electrodes for deep brain stimulation. For clinical translation, OCT can be integrated into standard operating equipment such as surgical microscopes (6, 7), and probes for stereotactic procedures (36). For the purposes of glioma surgery, the cross-polarization mode provides a good illustration of a functional extension to OCT, which can offer opportunities to improve the visual assessment of OCT data.

## Conclusion

CP OCT based on the visual assessment of obtained images is a promising imaging tool for distinguishing tumorous and non-tumorous tissues during glioma resection. The most powerful criteria are the signal intensities in co- and cross-polarization. Further to this purpose, additional criteria such as the homogeneity of intensity and the uniformity of attenuation can also be used. The simultaneous assessment of CP OCT images in co- and cross-polarization has demonstrated better diagnostic accuracy for distinguishing white matter from tumor tissue than OCT alone. Differentiation between cortex and white matter can be performed based on the criterion “loss of normal attenuation” (loss of the “typical view” of vertical striations on CP OCT images). In distinguishing white matter and tumors the diagnostic accuracy using the visual CP OCT criteria was 87–88%. The CP OCT data is easily associated with intraoperative neurophysiological and neuronavigation findings and can provide supplementary information for neurosurgeons during tumor resection.

## Data Availability

All datasets generated for this study are included in the manuscript and/or the supplementary files.

## Author Contributions

KY, EK, AM, and NG: study concept and design. KY, EG, PS, IM, and LK: data acquisition and quality control of data. KY, AM, SK, GG, and EK: data analysis and interpretation. KY, EK, AM, and NG: manuscript preparation. AP, EZ, and NG: manuscript review.

### Conflict of Interest Statement

The authors declare that the research was conducted in the absence of any commercial or financial relationships that could be construed as a potential conflict of interest.
